# Store-Operated Calcium Channels in Physiological and Pathological States of the Nervous System

**DOI:** 10.3389/fncel.2020.600758

**Published:** 2020-11-26

**Authors:** Isis Zhang, Huijuan Hu

**Affiliations:** Department of Anesthesiology, Rutgers New Jersey Medical School, Rutgers, The State University of New Jersey, Newark, NJ, United States

**Keywords:** store-operated calcium channels, STIM, Orai1, nervous system, neuron, glia, Alzheimer’s disease, pain

## Abstract

Store-operated calcium channels (SOCs) are widely expressed in excitatory and non-excitatory cells where they mediate significant store-operated calcium entry (SOCE), an important pathway for calcium signaling throughout the body. While the activity of SOCs has been well studied in non-excitable cells, attention has turned to their role in neurons and glia in recent years. In particular, the role of SOCs in the nervous system has been extensively investigated, with links to their dysregulation found in a wide variety of neurological diseases from Alzheimer’s disease (AD) to pain. In this review, we provide an overview of their molecular components, expression, and physiological role in the nervous system and describe how the dysregulation of those roles could potentially lead to various neurological disorders. Although further studies are still needed to understand how SOCs are activated under physiological conditions and how they are linked to pathological states, growing evidence indicates that SOCs are important players in neurological disorders and could be potential new targets for therapies. While the role of SOCE in the nervous system continues to be multifaceted and controversial, the study of SOCs provides a potentially fruitful avenue into better understanding the nervous system and its pathologies.

## Introduction

Store-operated calcium channels (SOCs) are calcium-selective cation channels that represent a major pathway for calcium signaling throughout the body. Due to their physical and functional connection to the endoplasmic reticulum (ER), SOCs play an important role in maintaining calcium homeostasis by inducing calcium entry after Ca^2+^ store depletion in the ER (Putney, 1986). This store-operated calcium entry (SOCE) drives a multitude of biological processes, including gene transcription, exocytosis, cell metabolism, and motility (Lewis, 2007). SOCs are composed of stromal interaction molecules (STIM1/2 proteins), which act as ER Ca^2+^ sensors, and Orai1/2/3 proteins, which form the structure of calcium release-activated calcium (CRAC) channels in the plasma membrane (Liou et al., 2005; Zhang et al., 2005; Gross et al., 2007; Wissenbach et al., 2007). Upon depletion of Ca^2+^ stores, STIM and Orai proteins migrate from their positions in the ER and PM, respectively, to form ER-PM junctions (Baba et al., 2003; Mercer et al., 2006). These junctions allow STIMs to bind to Orais, opening the channel to permit calcium entry. Reuptake of Ca^2+^ by sarco/ER Ca^2+^ ATPase (SERCA) leads to the end of SOCE, and STIMs and Orais return to their original locations (Alonso et al., 2012; Prakriya and Lewis, 2015). STIM2 also acts as a regulator in this mechanism, sensing a small drop of Ca^2+^ concentration in the cell and regulating basal cytosol and ER calcium level (Berna-Erro et al., 2009).

Neurons possess a variety of ion channels, receptors, transporters, and plasma membrane Ca^2+^ ATPase that work together to maintain Ca^2+^ homeostasis. In neurons, voltage-gated calcium channels (VGCCs) and ligand-gated cation channels were thought to be the primary channels involved in Ca^2+^ homeostasis. While SOCs are well recognized as the principal route of Ca^2+^ entry in non-excitable cells, SOCE was considered as a residual calcium entry in neurons and its function was neglected. Some early studies show that SOCs are not involved in [Ca^2+^]_i_ homeostasis/oscillations (Friel and Tsien, 1992; Nuñez et al., 1996). However, a growing body of evidence indicates that SOCs are also important in mediating Ca^2+^ influx in neurons from different brain regions, spinal cord, and dorsal root ganglion neurons (Emptage et al., 2001; Klejman et al., 2009; Gruszczynska-Biegala et al., 2011; Gao et al., 2013; Gruszczynska-Biegala and Kuznicki, 2013; Xia et al., 2014; Wei et al., 2017).

While Orais have been identified as key components of CRAC channels, the transient receptor potential (TRP) channels have also been suggested to be constituents of CRAC channels, in particular canonical TRP channel 1 (TRPC1) (Zhu et al., 1996; Zitt et al., 1996; Brough et al., 2001; Kim et al., 2009). TRPC1 has been shown to complex with STIM1 and Orai1 and have a role in the activation of SOCE (Ong et al., 2007; Cheng et al., 2008; Jardin et al., 2008; Nascimento Da Conceicao et al., 2019). However, TRPC channels’ role in calcium entry is controversial. For example, while some studies show TRPC1 contributing to SOCE, the TRPC1 mediated currents did not resemble I_CRAC_ and did not reproduce the biophysical properties of I_CRAC_ (Ambudkar et al., 2017; Lopez et al., 2020). As such, while much of the research focusing on SOCE studies the interaction between STIM1, Orai1, and TRPC isoforms, the true contribution of these channels to calcium entry remains contested. Furthermore, the closely related subfamilies TRPV (vanilloid) and TRPM (melastatin) have also recently been shown to have involvement in calcium influx via SOCE (Authi, 2007; Ma et al., 2011; Harisseh et al., 2013; Liu X. et al., 2017; Bastián-Eugenio et al., 2019). Interestingly, a recent study showed TRPM7 channel kinase modulated SOCE, which suggests that while the activity of the channels helps maintain calcium homeostasis at rest, it is smaller domains within the channel that truly control calcium flux (Faouzi et al., 2017).

STIM and Orai proteins are widely expressed throughout the body, having important roles in various physiological processes and involvement in the pathological conditions of the major organ systems (McCarl et al., 2009; Kiviluoto et al., 2011; Selvaraj et al., 2012; Collins et al., 2013; Sun S. et al., 2014; Lacruz and Feske, 2015; Avila-Medina et al., 2018; Zhang et al., 2020). SOCs have been well studied in non-excitable cells. In recent years, attention has turned to their role in neurons and glia. All homologs of STIM and Orai are present in murine and human brain tissues (Gross et al., 2007; Berna-Erro et al., 2009; Skibinska-Kijek et al., 2009; Gruszczynska-Biegala et al., 2011). STIM1 and STIM2 are differentially expressed among mouse and human brain regions (Lein et al., 2007). Regional distribution of Orai mRNAs in the brain has only been done in mice. In whole murine brain tissue, all three Orai family members are expressed, and Orai1 mRNA is less abundant than Orai2 and Orai3 mRNA (Gross et al., 2007; Lein et al., 2007; Takahashi et al., 2007). Orai2 is highly expressed in the spinal cord and DRG while Orai1 and Orai3 are expressed at a moderate and low level in the spinal cord and DRG, respectively (Xia et al., 2014; Wei et al., 2017; Dou et al., 2018). Orai1 protein is uniformly distributed throughout the human and rodent brain (Guzman et al., 2014). SOCs are functional in neurons and glia from different regions in the nervous system (Hartmann et al., 2014; Gao et al., 2016; Korkotian et al., 2017; Dou et al., 2018; Chen-Engerer et al., 2019; Stegner et al., 2019). Growing evidence shows that SOCs play an important role in neuronal signaling and plasticity, and have been implicated in neurological disorders (Kraft, 2015; Majewski and Kuznicki, 2015; Lu and Fivaz, 2016; Bollimuntha et al., 2017; Wu H. E. et al., 2018; Alvarez et al., 2020). In this review, we summarize the current understanding of the physiological role of SOCs in the nervous system by outlining their expression, molecular components, and functions in neurons from different brain regions and glial cells. We will also describe the multitude of pathologies that have been linked to dysregulation of SOCE in different types of nervous cells and discuss future directions of research.

## SOCs in the Cerebral Cortex

### SOC Components and Functions in Cortical Neurons

The mouse cortex and cortical neurons express both STIM mRNAs with STIM2 being the predominant isoform (Lein et al., 2007; Chauvet et al., 2016). In the human cortex, STIM1 protein was found in medium level in the cerebral cortex (Uhlén et al., 2015). The mRNA expression of Orais is controversial. Chauvet et al. (2016) have reported that Orai2 is highly expressed in the mouse cortical neurons while Orai1 and Orai3 mRNA levels are low or undetectable. Conversely, González-Sánchez et al. (2017) have shown that all three Orai isoforms are expressed in cortical neurons with Orai1 being the predominant isoform. Orai1 mRNA and protein expression in cortical neurons was also demonstrated in cortical neurons by other groups (Gruszczynska-Biegala and Kuznicki, 2013; Secondo et al., 2019). A recent study has further confirmed the expression of three *Orai* genes (Bouron, 2020). Orai3 is the major isoform expressed at the embryonic days 11 (E11) stage and remains constant during corticogenesis. Going from E11 to post-natal day 1 (PN1), Orai2 is upregulated the most, becoming the most expressed Orai gene at the end of corticogenesis and postnatally. Interestingly, Orai1 has the lowest rate of expression throughout corticogenesis (Bouron, 2020).

Both STIM proteins are involved in calcium homeostasis in cortical neurons, STIM1 mainly activates SOCE, whereas STIM2 regulates resting Ca^2+^ levels (Gruszczynska-Biegala and Kuznicki, 2013). Cortical SOCE induced by thapsigargin (TG), a Ca^2+^-ATPase inhibitor, is sensitive to Orai blockers, but not TRPC inhibitors (Chauvet et al., 2016), suggesting Orais mediate cortical SOCE. A recent study further confirmed that Orai2 is a major contributor to neuronal SOCE in the cortex (Stegner et al., 2019). STIM2 and Orai1 form hetero-complexes in rat cortical neurons in response to a low extracellular calcium level, which implies that these proteins regulate basal intracellular calcium levels in these neurons (Gruszczynska-Biegala and Kuznicki, 2013). Furthermore, calcium homeostasis is key to normal cortical neuron functions (Ma et al., 2016; Wegierski and Kuznicki, 2018). SOCE has been found to regulate neuronal calcium homeostasis during cortical development (Guner et al., 2017). Moreover, previous studies have shown that STIM1 is engaged by metabotropic glutamate receptors (mGluR) and is a key regulator of mGluR1 related Ca^2+^ signaling (Hou et al., 2015; González-Sánchez et al., 2017). As such, the regulation of long term depression of cortical neurons has been linked to SOCE (González-Sánchez et al., 2017).

### SOCs in Acquired Brain Injury

Ca^2+^ homeostasis alteration has been shown to contribute to secondary neuronal damage and altered physiology after traumatic brain injury (Weber, 2012). Given that cortical SOCs mediate Ca^2+^ entry and regulate calcium homeostasis, they may play a role in brain injury. A previous study has reported that STIM1 expression is significantly increased at both mRNA and protein levels following traumatic brain injury, and downregulation of STIM1 leads to increased preservation of neuronal viability and inhibition of apoptosis (Hou et al., 2015). Furthermore, STIM1 expression is increased in neurons in the early stages post diffuse axonal injury, indicating that abnormal SOCE may participate in Ca^2+^ overload of neurons (Li Y. et al., 2013). However, Rao et al. (2015) found that the expression of STIM2 but not STIM1 is increased in traumatic neuronal injury, and that downregulation of STIM2 but not STIM1 improved neuronal survival and preserved neurological function both *in vitro* and *in vivo*. The latter is consistent with a previous study that STIM2-deficient mice are protected from cerebral damage after ischemic stroke (Berna-Erro et al., 2009). More research into roles of STIMs in traumatic brain injury is therefore warranted.

Interestingly, in a rat model of ischemic stroke, Secondo et al. reported that STIM1 and Orai1 proteins were significantly decreased in the ipsilesional cortex. Similarly, STIM1 and Orai1 transcripts and proteins were also reduced after exposure of rat cortical neurons to oxygen and glucose deprivation for 3 h, leading to decreases in SOCE and CRAC currents (Secondo et al., 2019; La Russa et al., 2020). Silencing of STIM1 or Orai1 negated the effect of ischemic preconditioning, which would normally induce increased tolerance for ischemia, and lead to ER stress and increased neuronal death (Secondo et al., 2019), suggesting that Orai1-mediated SOCE is required for ischemic preconditioning. As such, STIM1 and Orai1 play a neuroprotective role post neural ischemic injury. In contrast to these findings, Stegner et al. (2019) found that Orai2 deficiency reduces Ca^2+^ accumulation and neuron death after exposure to oxygen and glucose deprivation and had a protective effect in a mouse model of ischemic stroke, indicating that disruption of this normal calcium response lowers calcium overload and thereby reduces neuronal damage in ischemic stroke. Although both Orai1 and Orai2 contribute to SOCE, they play distinct roles in ischemic stroke. As such, further research into the mechanisms and effects of calcium flux in cortical neurons is essential in understanding their roles in stroke.

## SOCs in the Hippocampus

### SOC Components and Functions in Hippocampal Neurons

The hippocampus has high STIM2 expression and low STIM1 level Both STIM1 and STIM2 mediate SOCE in hippocampal neurons (Zhang et al., 2014, 2015; Ryazantseva et al., 2018). In hippocampal CA1 tissue, all three Orai homologs are expressed while Orai2 is expressed at the highest level (Chen-Engerer et al., 2019). Orai2 is selectively involved in IP_3_ sensitive calcium stores in the soma of CA1 pyramidal neurons (Chen-Engerer et al., 2019), suggesting an important role of Orai2 in neuronal SOCE in the CA1 region. Interestingly, Orai1 was found in a large proportion of dendritic spines while Orai2 was detected mainly in dendritic shafts but to a lesser extent in spines (Korkotian et al., 2017; Tshuva et al., 2017). The different spatial distribution of Orai1 and Orai2 may indicate distinct roles in hippocampal function.

Store-operated calcium entry is an important Ca^2+^ influx pathway and plays an important role in basic neuronal functions and Ca^2+^ homeostasis in hippocampal neurons (Emptage et al., 2001; Baba et al., 2003). Samtleben et al. (2015) found that free ER and cytosolic calcium in hippocampal neurons was lost continuously across the plasma membrane under transiently calcium-free conditions. Interestingly, when SOCE was inhibited, an immediate decline in ER calcium was observed, suggesting that SOCs counteract continuous loss of ER and cytosolic calcium and maintain basal Ca^2+^ levels in hippocampal neurons (Samtleben et al., 2015). In addition, Orai1 is preferentially localized in spines and helps regulate spine plasticity (Korkotian et al., 2014; Segal and Korkotian, 2016). This finding aligns with data that Orai1 plays a key role in synapse formation, maturation, and plasticity (Korkotian et al., 2017; Tshuva et al., 2017). Furthermore, STIM2 facilitates synaptic delivery and regulates activity-dependent changes in synaptic strength (Yap et al., 2017).

### SOCs in Epilepsy and Alzheimer’s Disease

Due to their important role in Ca^2+^ signaling and neuronal plasticity, SOCs have been implicated in diseases related to hippocampal dysfunction. Steinbeck et al. (2011) found that STIM1 and STIM2 expression was increased in a rat model of chronic epilepsy. In hippocampal specimens from medial temporal lobe epilepsy patients, STIM1 and STIM2 were also elevated (Steinbeck et al., 2011). Pharmacologic inhibition of SOCE suppressed interictal spikes and rhythmizing epileptic burst activity (Steinbeck et al., 2011). Interestingly, Orai1 overexpression has also been found to cause seizure like events in aged female mice, suggesting that SOC dysfunction on its own is a potential etiology for seizure and epilepsy (Maciąg et al., 2019; Majewski et al., 2019).

Alzheimer’s disease (AD) is a chronic neurodegenerative disease and the most common cause of dementia worldwide (Lane et al., 2018). AD has been linked to dysregulation of SOCs in hippocampal neurons (Raza et al., 2007; Popugaeva et al., 2015b). In long-term cultures reflecting aging neurons, there is remodeling of Ca^2+^ influx and efflux with downregulation of STIM1 and Orai1, increased expression of the mitochondrial Ca^2+^ uniporter and Ca^2+^ stores in these neurons, and enhanced Ca^2+^ release (Calvo-Rodríguez et al., 2016; Calvo-Rodriguez et al., 2020). Reduction in STIM1 expression has also been found in brain tissues of pathologically confirmed AD patients (Pascual-Caro et al., 2018). Furthermore, neuronal SOCE in postsynaptic spines plays a key role in stability of mushroom shaped “memory spines,” the loss of which are indicative of a deficiency in synaptic communication (Sun S. et al., 2014; Ryskamp et al., 2019). This aligns with data that show in a murine model of AD, overexpression of STIM2 attenuates Aβ42 oligomers-induced mushroom spine loss *in vitro* and *in vivo* through maintenance of normal SOCE (Popugaeva et al., 2015a; Zhang et al., 2015, 2016). Presenilin 1 and presenilin 2 have been associated with Familial AD, another form of AD (Lanoiselée et al., 2017). Interestingly, in the PSEN1ΔE9 mutation model of Familial AD, there is an increase in SOCE in postsynaptic spines in primary hippocampal cultures. Pharmacologic inhibition of SOCE rescued mushroom spine loss in hippocampal neurons (Chernyuk et al., 2019). These studies suggest that SOCs play a distinct role in different forms of AD.

## SOCs in the Striatum

### SOC Components and Functions in Striatal Medium Spiny Neurons (MSNs)

Western blot analysis revealed that the SOC proteins are expressed in medium spiny neurons (MSNs) (Wu J. et al., 2018). The molecular composition of SOCs in MSNs has not been well established. Kikuta et al. (2019) found strong expression of Orai2, moderate expression of Orai3, and sparse expression of Orai1 in striatal GABAergic neurons with strong expression of both STIM1 and STIM2. Interestingly, knockdown of STIM1, Orai1, or TRPC1 proteins leads to dramatic reduction of SOCE in MSNs (Vigont et al., 2015), indicating STIM1, Orai1, and TRPC1 mediate SOCE in MSNs. Additionally, a recent study demonstrates that deletion of TRPC1 largely reduced SOCE in MSNs (Wu J. et al., 2018), further indicating an important role of TRPC1 in MSN SOCE. In addition, SKF96365, a SOC and TRPC channel inhibitor, was found to reduce the frequency of spontaneous slow Ca^2 +^ oscillations in these neurons, indicating the SOCE has a role in Ca^2 +^ signaling in MSNs (Kikuta et al., 2019).

### SOCs in Huntington’s Disease (HD)

Huntington’s disease (HD) is the most common monogenic neurodegenerative disease (Ghosh and Tabrizi, 2018). Patients develop motor, cognitive, and psychiatric symptoms in middle age, with continuous neurodegeneration until the end of their lives (Ghosh and Tabrizi, 2018; McColgan and Tabrizi, 2018). Mutant Huntingtin (mHtt) protein causes striatal neuron dysfunction, synaptic loss, and ultimately neurodegeneration in HD (McColgan and Tabrizi, 2018). Neuronal cells expressing mHtt show inhibition of the SOC pathway through binding of mHtt to the type 1 inositol (1,4,5)-trisphosphate receptor (InsP_3_R1), which increases the receptor’s sensitivity to activation by InsP_3_ (Wu et al., 2011; Vigont et al., 2014). The overactivity of this pathway is also in part because STIM2 expression is elevated in these neurons, which leads to further dysregulation of SOCE and spine loss in MSNs (Wu et al., 2016). Additionally, knockdown of TRPC1, TRPC6, Orai1, or Orai2 also shows protective effects on medium spiny neuron spines in HD model mice (Wu J. et al., 2018). As such, SOCs have been studied as a potential therapeutic target for HD, with the effect of the potential anti-HD drug EVP4593 on calcium regulation via these channels being investigated in recent years (Wu et al., 2016; Vigont et al., 2018).

## SOCs in the Substantia Nigra

### SOC Components and Functions in Dopaminergic (DA) Neurons

The protein expression of STIM1, Orai1, and several TRPC channels was observed and robust SOCE and SOC currents were recorded in dopaminergic (DA) neurons (Selvaraj et al., 2012; Sun et al., 2018). While the molecular identity of SOCE has not been conclusively identified in DA neurons, it has been reported that downregulation of STIM1 or TRPC1 leads to the loss of SOCE (Selvaraj et al., 2012; Sun et al., 2018), suggesting that SOCE is mediated by STIM1 and TRPC1 in DA neurons in the substantia nigra (Selvaraj et al., 2012).

### SOCs in Parkinson’s Disease

Parkinson’s disease is defined by death of DA neurons in the substantia nigra, which leads to the degeneration of motor skills and memory that is characteristic of the disease (Reich and Savitt, 2019). Ca^2+^ entry is crucial in regulation of mitochondrial oxidative phosphorylation in DA neurons (Surmeier et al., 2017). Interestingly, Ca^2+^ entry also drives basal mitochondrial oxidant stress in these neurons (Surmeier et al., 2017). SOCE in DA neurons regulates Ca^2+^ entry and activates the AKT/mTOR pathway, a known neuroprotective pathway in Parkinson’s disease (Selvaraj et al., 2012). Additionally, in normal conditions, pacemaking activity in DA neurons is inhibited by the TRPC1-STIM1 complex (Sun et al., 2017). When neurotoxins mimicking Parkinson’s disease were introduced in DA neurons, TRPC1 expression was targeted, increasing activity of L-type Ca^2+^ channels and caspases, leading to neurodegeneration (Sun et al., 2017). Furthermore, postmortem substania nigra samples from Parkinson’s disease individuals also showed decreased TRPC1 expression in the substantia nigra pars compacta region compared to non-Parkinson’s disease individuals (Selvaraj et al., 2012).

## SOCs in the Cerebellum

### SOC Components and Functions in Purkinje and Granule Neurons

The mouse cerebellum expresses the highest level of STIM1 among all brain regions (Lein et al., 2007; Hartmann et al., 2014). In a human brain tissue study, STIM1 protein expression was found to be relatively high in the cerebellum (Uhlén et al., 2015). In mouse Purkinje neurons, STIM1 expression was more robust than that of STIM2 (Hartmann et al., 2014). All three Orai isoforms are detectable in the cerebellum and in Purkinje neurons, with Orai2 as the dominant isoform in Purkinje neurons (Hartmann et al., 2014). Interestingly, research has focused on STIM1 and Orai1 proteins, which appear to mediate SOCE in Purkinje neurons (Klejman et al., 2009). In Purkinje neurons, STIM1 controls glutamate receptor-dependent synaptic transmission and motor learning in mice (Hartmann et al., 2014). Ryu et al. (2017) found deletion of STIM1 delayed clearance of cytosolic Ca^2+^ during ongoing neuronal firing, reduced Purkinje neuronal excitability, and impaired intrinsic plasticity without affecting long term synaptic plasticity. Cerebellar granule cells also express STIM1 and SOCs are functional in these neurons (Singaravelu et al., 2008; Klejman et al., 2009). Although essential components of the SOCE pathway are not well characterized in granule cells, expression and pharmacological studies suggest that STIM1 and Orai1 may mediate granule SOCE (Singaravelu et al., 2008; Klejman et al., 2009).

### SOC in Motor Memory Consolidation

As discussed above, SOCs are important for maintaining cellular function in Purkinje cells. Defects in intrinsic plasticity of Purkinje neurons can lead to formation of aberrant neural plasticity in vestibular nucleus neurons, and thereby inhibition of SOCE can affect long-term storage of motor memory and lead to deficits in motor skills (Jang et al., 2020). Furthermore, STIM1 knockout mice showed severe memory consolidation deficiency in vestibule-ocular reflex memory (Ryu et al., 2017). As intrinsic plasticity emerges as an important factor in information processing and memory formation, especially in Purkinje neurons, SOCE and its modulation of intrinsic plasticity may elucidate potential etiologies for motor abnormalities and memory loss (Shim et al., 2018). For example, given that impaired intrinsic plasticity or degeneration of Purkinje neurons is associated with ataxia and STIM1 knockdown is linked to impaired intrinsic plasticity (Ryu et al., 2017; Ady et al., 2018; Hoxha et al., 2018), deficiency of STIM1-mediated SOCE could be a potential cause of ataxia.

## SOCs in the DRG and Spinal Cord

### SOC Components and Functions in Spinal Cord Dorsal Horn and DRG Neurons

The spinal cord dorsal horn and dorsal root ganglia (DRG) act together to relay sensory information from the periphery to the CNS (Cho, 2015). We and others have demonstrated that Orai1/2/3 and STIM1/2 are expressed in dorsal horn neurons, with STIM1, STIM2, and Orai1 acting as key mediators of SOCE (Guzman et al., 2014; Xia et al., 2014). In addition, we have shown that activation of Orai1 increases neuronal excitability and reduces A-type potassium channels in dorsal horn neurons (Dou et al., 2018).

We and others have also shown that the SOC proteins are also expressed in DRG (Gemes et al., 2011; Wei et al., 2017). STIM1, STIM2, Orai1, and Orai3 mediate SOCE in DRG neurons, with small and medium sized DRG neurons exhibiting more robust SOCE after Ca^2+^ depletion by TG (Wei et al., 2017). In particular, nociceptors including TRPV1-, TRPA1-, TRPM8-, and IB4-positive DRG neurons displayed greater SOCE than non-nociceptive neurons. In addition, in nociceptive DRG neurons, activation of SOCs by TG increases neuronal excitability while Orai1 and Orai3 double knock down abolished such effect (Wei et al., 2017), suggesting SOCE is an important Ca^2+^ influx pathway for nociceptors.

### SOCs in Pain

While the role of SOCs in pain is not completely understood, there is strong evidence that SOCs play an important role in modulating nociception and chronic pain (Gao et al., 2013, 2015; Qi et al., 2016). We have reported that pretreatment with YM-58483, a potent SOC inhibitor, reduced acute pain and prevented the development of CFA- or collagen-induced inflammatory pain. YM-58483 also attenuated thermal and mechanical hypersensitivity after inflammatory pain was established (Gao et al., 2013, 2015). Moreover, administration of YM-58483 diminished neuropathic pain induced by spared nerve injury, a well-established neuropathic pain model (Gao et al., 2013). Consistent with the pharmacological results, Orai1 deficiency significantly decreased acute pain induced by noxious stimuli, reduced intraplantar carrageenan injection-induced pain, and abolished the increase in neuronal excitability induced by TG (*in vitro*) and intraplantar carrageenan injection (recorded in spinal cord slices) (Dou et al., 2018). These data suggest that SOCE is an important player in nociception and inflammatory pain, and could potentially be used as a novel target for chronic pain.

## SOCs in Glial Cells

Glial cells play essential roles in brain homeostasis. There are three main types of glia in the CNS: astrocytes, microglia, and oligodendrocytes; all play distinct roles supporting neurons and their interconnections (Jessen, 2004). For astrocytes and microglia, the regulation of their activities is in part controlled by Ca^2+^ signaling and their own calcium homeostasis (Kettenmann et al., 2011; Shigetomi et al., 2019). The various immune pathways mediated by SOCE in astrocytes and microglia point to the importance of SOCs in modulating inflammation and CNS defense, as well as identify dysfunction of SOCs as potential causes for diseases of abnormal immunity and inflammation in the CNS.

### SOC Components and Functions in Astrocytes

The expression of STIM1/2 and Orai1/2/3 in astrocytes has been demonstrated by multiple groups (Jung et al., 2000; Lo et al., 2002; Singaravelu et al., 2006; Moreno et al., 2012; Gao et al., 2016; Kwon et al., 2017). It is well documented that SOCE can be induced in astrocytes in the central nervous system. In hippocampal astrocytes, CRAC channels regulate astrocyte Ca^2+^ signaling, gliotransmitter release, and astrocyte-mediated tonic inhibition of CA1 pyramidal neurons (Toth et al., 2019). In cortical astrocytes, STIM1 in combination with Orai1 and Orai3 mediates SOCE in a majority of cells (Moreno et al., 2012; Kwon et al., 2017). Similarly, in spinal astrocytes, STIM1, STIM2, and Orai1 were identified as primary mediators of SOCE (Gao et al., 2016). Retinal Müller glia also express STIMs and Orais (Molnar et al., 2016). Double labeling results show that STIM1 (not STIM2) is predominantly found in Müller glia. Interestingly, SOCE is mediated by synergistic activation of TRPC and Orai channels in these cells (Molnar et al., 2016). Furthermore, a group developed a mathematical model for calcium flux in astrocytes, and reported that while Ca^2+^ influx levels through SOCs in astrocytes are low, sustained calcium oscillations require SOC activation (Handy et al., 2017). As the intrinsic frequency of calcium oscillations is theorized to be important in regulating activities such as gliotransmission, SOCs could be used as a potential modulator of these activities (De Pittà et al., 2009; Handy et al., 2017).

### SOC Components and Functions in Microglia

Microglial SOCE was first reported in mice (Toescu et al., 1998). Later studies have reported that SOCE is present in human microglia (Wang et al., 1999; McLarnon et al., 2000; Khoo et al., 2001; Hong et al., 2006). Multiple studies have demonstrated STIM1/2 and Orai1/2/3 are expressed and SOCE occurs in these cells (Kraft, 2015; Michaelis et al., 2015; Gilbert et al., 2016). STIM1 and Orai1 play a major role in microglial SOCE and SOC currents while STIM2 is less effective to activate SOCE than STIM1 (Michaelis et al., 2015; Lim et al., 2017). Functionally, SOC inhibition or ablation of STIM1, STIM2, or Orai1 has been shown to inhibit migration, phagocytosis, cytokine secretion, and NFAT1 activity (Ikeda et al., 2013; Heo et al., 2015; Michaelis et al., 2015; Lim et al., 2017).

### Glial SOCs in CNS Diseases

Reactive astrocytes have been implicated in many CNS disorders, such as epilepsy, Alzheimer disease, Parkinson’s disease, and multiple sclerosis (Glass et al., 2010; Brambilla et al., 2013; Devinsky et al., 2013). Spinal astrocytes have been recognized as active participants in chronic pain conditions (Ji et al., 2006; McMahon and Malcangio, 2009). We have shown that activation of SOCs increases TNF-α and IL-6 production, while knockdown of STIM1 or Orai1 greatly attenuates cytokine production (Gao et al., 2016). Furthermore, knockdown of STIM2 and Orai1 decreases lipopolysaccharide-induced TNF-α and IL-6 production without altering viability of astrocytes (Gao et al., 2016). These data suggest that SOCs may represent potential therapeutic targets for neuroinflammation.

Store-operated calcium entry has been linked to the pathogenesis of glioblastoma multiforme (GBM), the highest grade glioma and most malignant astrocytoma (Motiani et al., 2013). In human glioblastoma cells, SOC function is largely enhanced compared to normal astrocytes (Kovacs et al., 2005). Multiple groups have found STIM1 and Orai1 knockdown lead to a dramatic decrease in cell invasion in GBM, as such STIM1 has been proposed as a potential target in GBM treatment (Liu et al., 2011; Li G. et al., 2013; Motiani et al., 2013). Interestingly, in a recent study, induction of SOCE suppressed GBM growth via inhibition of Hippo pathway transcriptional coactivators YAP/TAZ (Liu et al., 2019). Hence, more research is warranted to establish the role of SOCs in GBM. Astrocyte Ca^2+^ activity also plays an important role in Rett syndrome disease progression. Dong et al. (2018) reported that spontaneous calcium activity is abnormal in RTT astrocytes *in vitro* and *in vivo*, which is caused by abnormal SOCE partially associated with elevated expression of TRPC4.

Microglial SOCE has been implicated in a variety of nervous system disorders as well. For example, AD microglia have significantly higher basal Ca^2+^ relative to microglia from non-AD people, and ATP- and PAF-induced SOCE is markedly reduced (McLarnon et al., 2005), indicating that microglia from AD patients have significant abnormalities in Ca^2+^ mediated signal transduction. Microglial SOCs may also play a role in PD. Lu et al. (2019) used 1-methyl4-phenylpyridinium (MPP), a metabolite of 1-Methyl-4-phenyl-1,2,3,6-tetrahydropyridine (MPTP, a mouse model of PD), to induce microgliosis and neuroinflammation and observed time-dependent upregulation of Orai1 and an increase of SOCE. A recent report suggests that microglial SOCs are also involved in brain trauma. In a mouse model of brain trauma, inhibition of SOCs decreases lesion size, brain hemorrhage, and improves neurological deficits associated with decreased microglial activation, and expression levels of iNOS, Orai1 and STIM1 (Mizuma et al., 2019). Moreover, in a model of helminth infection, there was negative regulation of TRPC1 and Orai1 mediated SOCE, which lead to inhibition of NF-κB and MAPK pathways in microglia (Sun Y. et al., 2014).

## Development of Potential Therapies

Store-operated calcium channels have been proposed as therapeutic drug targets for cancer, autoimmune, and inflammatory disorders (Stauderman, 2018; Feske, 2019; Khan et al., 2020). Great efforts have been made to identify potent and selective Orai1 inhibitors. Several compounds have entered clinical trials (Stauderman, 2018). However, challenges have been encountered in developing CRAC channel blockers that have high selectivity and low side effects due to their expression profile across major organ systems (Liu S. et al., 2017). While preclinical studies continue to identify more potent and selective compounds, new chemical scaffolds that target different components or new pharmacophores in the SOC complex may offer great opportunities to develop better therapeutics. Drug development is even more challenging for treatments of CNS diseases because of the nervous system’s complex and poor translation from animal models to human disease. As such, drug discovery of SOC inhibitors for CNS disorders is still in the early phase of target validation. To validate SOCs as therapeutic targets for these diseases, we should also consider whether existing therapies modulate SOCs. It has been reported that non-steroidal anti-inflammatory drugs (NSAIDs) sulindac, salicylate, and other NSAIDs including ibuprofen and indomethacin have been found to inhibit SOCE in colon cancer cells and in vascular smooth muscle cells (Muñoz et al., 2011; Hernández-Morales et al., 2017; Villalobos et al., 2019). It would be worthy to investigate whether these existing drugs can treat CNS disorders associated with neuroinflammation. Elucidating how these treatments interact with SOCs can lend evidence to SOC modulators as potential treatments in these pathologies.

## Discussion

**FIGURE 1:**
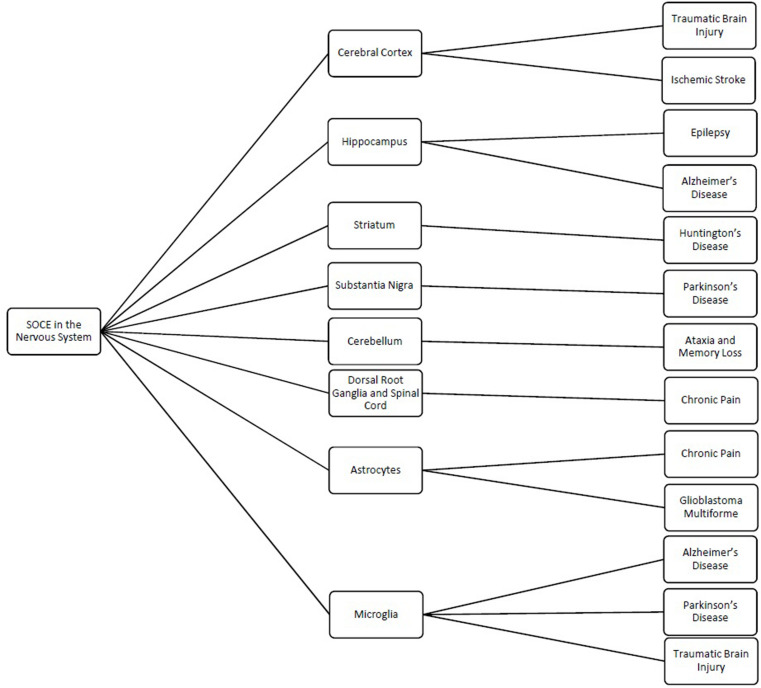
Schematic showing the different regions and cells of the nervous system where store-operated calcium entry (SOCE) has been shown to regulated cellular processes and the related pathologies linked to dysfunction of SOCE in these regions.

Store-operated calcium channels are functional throughout the nervous system and regulate a wide variety of physiological processes (Table 1). While STIMs and Orais have been shown to have significant expression in many regions of the nervous system, interestingly their expression levels and distribution patterns vary during developmental phases and in different regions of the nervous system. *Stim1* is robustly expressed in Purkinje neurons (Hartmann et al., 2014), but *Stim2* is more abundant in hippocampal neurons (Berna-Erro et al., 2009). STIM1 mainly activates SOCE, whereas STIM2 is more involved in regulation of basal intracellular calcium levels (Gruszczynska-Biegala and Kuznicki, 2013; Xia et al., 2014). *Orais* are differentially expressed in different cell types from the nervous system with *Orai2* being the predominant isoform. Orai2 has been reported to contribute to neuronal SOCE in the cortex and hippocampus while Orai1 is the major functional components responsible for SOCE in most cell types. In contrast, Orai3 appears to play a less important role. Furthermore, the various interplays of the TRP family with STIM and Orai proteins complicate the picture. As such, it is not surprising that while SOCE has been implicated in many neuronal processes from developmental signaling to pain transmission, its role in each of these actions is not consistent. Thus, it is also not surprising that loss- and gain-of-SOCE and thereby disruption of calcium homeostasis is implicated in such a wide range of neurological diseases (Figure 1). Further research into the specific expression and factors influencing SOCE in each of the aforementioned nervous system cells is warranted to elucidate their true roles in these physiological and pathological processes and to clarify whether their putative potential as treatments in these pathologies is valid.

**TABLE 1:** A summary table of the various nervous cells demonstrating SOCE and their SOCE-related physiological functions.

**Cell**	**SOCE-related physiological functions**	**References**
Cortical neurons	Ca^2+^ homeostasis during cortical development and in developed neurons Long term depression Long term potentiation	Gruszczynska-Biegala and Kuznicki, 2013; Garcia-Alvarez et al., 2015; Hou et al., 2015; González-Sánchez et al., 2017; Guner et al., 2017; Bouron, 2020
Hippocampal neurons	Counteract continuous loss of Ca^2+^ across the plasma membrane to maintain basal Ca^2+^ homeostasis Synapse formation, maturation, and plasticity	Samtleben et al., 2015; Segal and Korkotian, 2016; Korkotian et al., 2017; Yap et al., 2017
Striatal medium spiny neurons	Involvement in spontaneous slow Ca^2+^ oscillations	Kikuta et al., 2019
Dopaminergic neurons	Regulation of mitochondrial oxidative phosphorylation Activation of AKT/mTOR pathway	Selvaraj et al., 2012; Sun et al., 2017; Surmeier et al., 2017
Purkinje neurons	Clearance of cytosolic Ca^2+^ during neuronal firing Modulation of neuronal excitability and intrinsic plasticity Refilling of calcium stores required for TRPC3 function Regulation of mGluR1/TRPC3-dependent slow excitatory synaptic potentials Cerebellar motor function	Hartmann et al., 2014; Ryu et al., 2017; Shim et al., 2018; Jang et al., 2020
Cerebellar granule cells	Involvement in spontaneous Ca^2+^ oscillations	Singaravelu et al., 2008
Spinal cord dorsal horn	Regulation of resting calcium homeostasis, A type potassium channels, and neuronal excitability	Xia et al., 2014
Dorsal root ganglion neurons	Modulation of neuronal excitability	Wei et al., 2017
Astrocytes	Gliotransmitter release/gliotransmission Tonic inhibition of CA1 pyramidal neurons Cytokine secretion	Handy et al., 2017; Toth et al., 2019
Muller glia	Depletion dependent Ca^2+^ homeostasis	Molnar et al., 2016
Microglia	Cellular migration Phagocytosis Cytokine secretion NFAT1 activity	Ikeda et al., 2013; Heo et al., 2015; Michaelis et al., 2015; Lim et al., 2017

As the field of SOCs in neurological diseases is relatively nascent, data are limited and understanding of the role of SOCs in excitable cells is continuously evolving. While there are data exhibiting SOC function and activation, many of these experiments have employed the use of exogenous chemicals to activate or inhibit these channels (Chen et al., 2013; Gao et al., 2015; Qi et al., 2016; González-Sánchez et al., 2017; Domenichini et al., 2018). Moreover, these channels are often studied *in vitro*. When placed in a physiologic setting without these exogenous chemicals, SOCs may not act as expected, which could complicate our understanding of their physiological role. As there is limited data on how SOCs are activated under physiological conditions and how they affect pathological conditions *in vivo*, further studies must be done to address these questions using animal models and genetic tools.

Given the importance of Ca^2+^ homeostasis and Ca^2+^ signaling throughout the nervous system, it is understandable that SOCs show connections to the wide variety of neurological disorders discussed throughout this review. Again, as data on SOCs in neurons and glial cells are limited, our understanding of where SOCs fit into the pathophysiology of dementia, pain, and other neurological disorders is ever evolving. In addition, SOCs are often discussed as potential novel targets for these neurological diseases (Wu et al., 2011; Gao et al., 2013; Cui et al., 2017; Stegner et al., 2019; Waldron et al., 2019). While data of modulating SOC function in animal models of diseases are promising, they cannot be taken in isolation. With STIM1/2 and Orai1/2/3 expressed widely throughout all body systems, potential therapies based on SOCE modulation must take into account systemic effects when evaluating efficacy and safety. Furthermore, with data showing upregulation or maintenance is beneficial in certain disease states but deleterious or even causative in others, it is important to consider potential therapies in context of other diseases during development.

In short, the study of SOCs has yielded a new perspective by which researchers can examine the nervous system and its pathologies. Although the molecular components of SOCE and their functional significance in the nervous system are still controversial and multifaceted, it is clear that SOCs play an important role in neuronal development, homeostasis, signaling, neuronal excitability, and synapse formation. Increasing evidence indicates that dysfunction of SOCs is linked to brain injury, epilepsy, AD, HD, and pain. Furthermore, by elucidating new modalities by which neurological pathologies arise, the study of SOCs will provide additional avenues for developing therapies for difficult to treat or incurable neurological disorders. While understanding of SOCs is still developing, there is tremendous potential for discovery and advancement of neuroscience through continued research in SOCs.

## Author Contributions

IZ prepared the manuscript. Both authors edited and approved the final manuscript.

## Conflict of Interest

The authors declare that the research was conducted in the absence of any commercial or financial relationships that could be construed as a potential conflict of interest.
